# *catena*-Poly[[[aqua­(glycine-κ*O*)lithium]-μ-glycine-κ^2^ *O*:*O*′] bromide]

**DOI:** 10.1107/S1600536812050660

**Published:** 2012-12-19

**Authors:** T. Balakrishnan, K. Ramamurthi, J. Jeyakanthan, S. Thamotharan

**Affiliations:** a School of Physics, Bharathidasan University, Tiruchirappalli 620 024, India; b Department of Physics and Nanotechnology, SRM University, Kattankulathur 603 203, India; c Department of Bioinformatics, Alagappa University, Karaikudi 630 003, India; d Department of Bioinformatics, School of Chemical and Biotechnology, SASTRA University, Thanjavur 613 401, India

## Abstract

In the title coordination polymer, {[Li(C_2_H_5_NO_2_)_2_(H_2_O)]Br}_*n*_, the Li^+^ cation is coordinated by three carboxyl­ate O atoms of zwitterionic glycine mol­ecules and by a water mol­ecule, forming a distorted tetra­hedral geometry. One of the two glycine mol­ecules bridges neighbouring complexes, forming an infinite chain parallel to the *c* axis. Polymeric chains are further linked by extensive hydrogen bonds involving the Br^−^ anions and glycine and water mol­ecules, producing a three-dimensional network.

## Related literature

For hydrogen-bonding motifs, see Bernstein *et al.* (1995 ▶). For glycine polymorphs, see: Marsh (1958 ▶); Iitaka (1960 ▶, 1961 ▶). For glycine with halogen and metal halogenides, see: Fleck (2008 ▶). For related structures, see: Müller *et al.* (1994 ▶); Baran *et al.* (2003 ▶, 2009 ▶); Fleck & Bohatý (2004 ▶); Fleck *et al.* (2006 ▶). For head-to-tail hydrogen bonds, see: Sharma *et al.* (2006 ▶); Selvaraj *et al.* (2007 ▶).
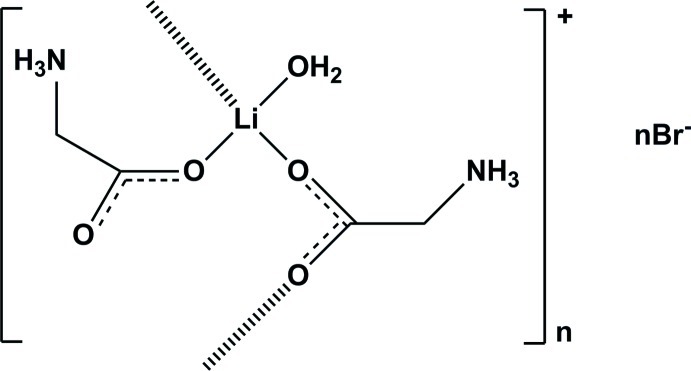


## Experimental

### 

#### Crystal data

[Li(C_2_H_5_NO_2_)_2_(H_2_O)]Br
*M* *_r_* = 255.01
Monoclinic, 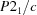

*a* = 7.5396 (6) Å
*b* = 17.4173 (14) Å
*c* = 8.2726 (12) Å
β = 118.138 (7)°
*V* = 957.96 (18) Å^3^
*Z* = 4
Mo *K*α radiation
μ = 4.28 mm^−1^
*T* = 173 K
0.61 × 0.30 × 0.30 mm

#### Data collection

STOE IPDS diffractometer
Absorption correction: multi-scan (*MULscanABS* in *PLATON*; Spek, 2009 ▶) *T*_min_ = 0.217, *T*_max_ = 0.277
7515 measured reflections
1847 independent reflections
1520 reflections with *I* > 2σ(*I*)
*R*_int_ = 0.043

#### Refinement

*R*[*F* ^2^ > 2σ(*F* ^2^)] = 0.021
*wR*(*F* ^2^) = 0.051
*S* = 0.96
1847 reflections
151 parameters
2 restraints
H atoms treated by a mixture of independent and constrained refinement
Δρ_max_ = 0.55 e Å^−3^
Δρ_min_ = −0.26 e Å^−3^

### 

Data collection: *EXPOSE* in *IPDS* (Stoe & Cie, 2000 ▶); cell refinement: *CELL* in *IPDS*; data reduction: *INTEGRATE* in *IPDS*; program(s) used to solve structure: *SHELXS97* (Sheldrick, 2008 ▶); program(s) used to refine structure: *SHELXL97* (Sheldrick, 2008 ▶); molecular graphics: *PLATON* (Spek, 2009 ▶); software used to prepare material for publication: *SHELXL97*.

## Supplementary Material

Crystal structure: contains datablock(s) I, global. DOI: 10.1107/S1600536812050660/aa2077sup1.cif

Structure factors: contains datablock(s) I. DOI: 10.1107/S1600536812050660/aa2077Isup2.hkl

Additional supplementary materials:  crystallographic information; 3D view; checkCIF report

## Figures and Tables

**Table 1:** Hydrogen-bond geometry (Å, °)

*D*—H⋯*A*	*D*—H	H⋯*A*	*D*⋯*A*	*D*—H⋯*A*
N1—H1*A*⋯O4^i^	0.94 (3)	1.83 (3)	2.774 (2)	176 (3)
N1—H1*B*⋯O1*W* ^ii^	0.92 (4)	2.15 (4)	2.989 (3)	151 (2)
N1—H1*C*⋯Br1^iii^	0.86 (3)	2.61 (3)	3.353 (2)	146 (3)
N2—H2*A*⋯Br1	0.81 (3)	2.48 (3)	3.283 (2)	170 (3)
N2—H2*B*⋯O1^iv^	0.90 (3)	2.00 (3)	2.833 (3)	153 (2)
N2—H2*C*⋯O1^v^	0.93 (3)	1.92 (3)	2.797 (2)	157 (3)
O1*W*—H1⋯O2^vi^	0.82 (2)	1.88 (2)	2.692 (2)	172 (3)
O1*W*—H2⋯Br1^vii^	0.83 (2)	2.48 (2)	3.2923 (17)	169 (3)

Symmetry codes: (i) 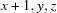; (ii) 
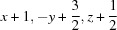
; (iii) 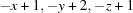; (iv) 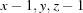; (v) 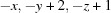; (vi) 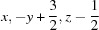; (vii) 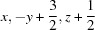.

**Fig. 1.**
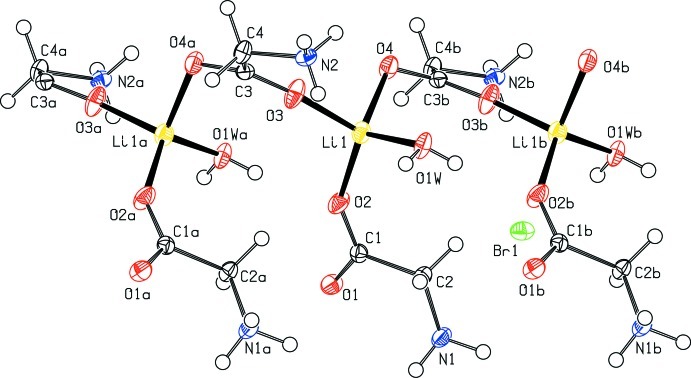
A view of the molecular structure of the title complex, showing the atom-labeling. Displacement ellipsoids are drawn at the 50% probability level. Symmetry codes: (a) x, -y+3/2, z-1/2 (b) x, -y+3/2, z+1/2.

**Fig. 2.**
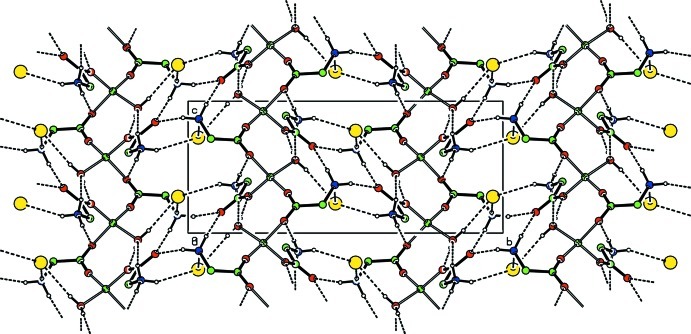
A view along the a axis of the crystal packing of the title complex. The hydrogen bonds are shown as dashed lines (see Table 1 for details). For clarity, H atoms not involved in hydrogen bonds have been omitted in this and subsequent figures..

**Fig. 3.**
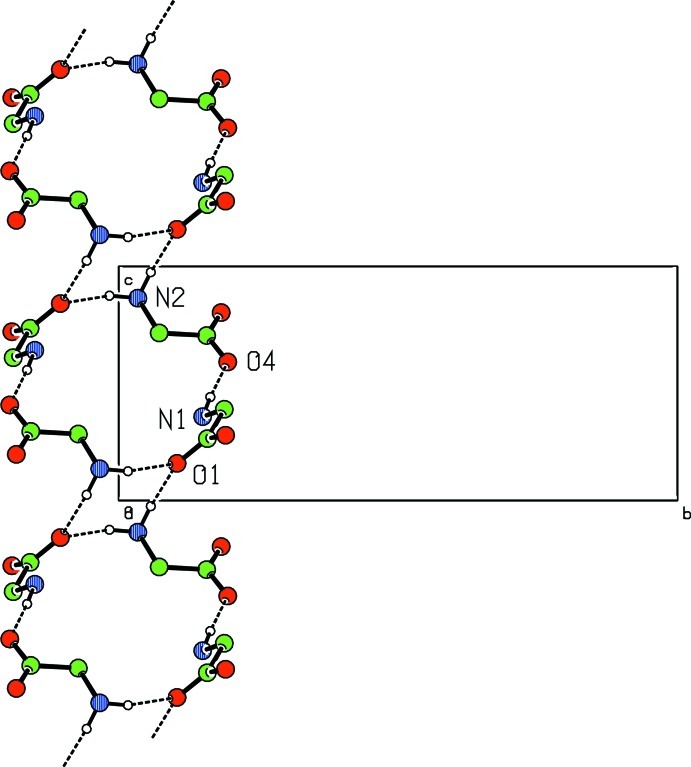
A partial view of the crystal structure of the title complex, showing the hydrogen bonds involving the glycine molecules.

**Fig. 4.**
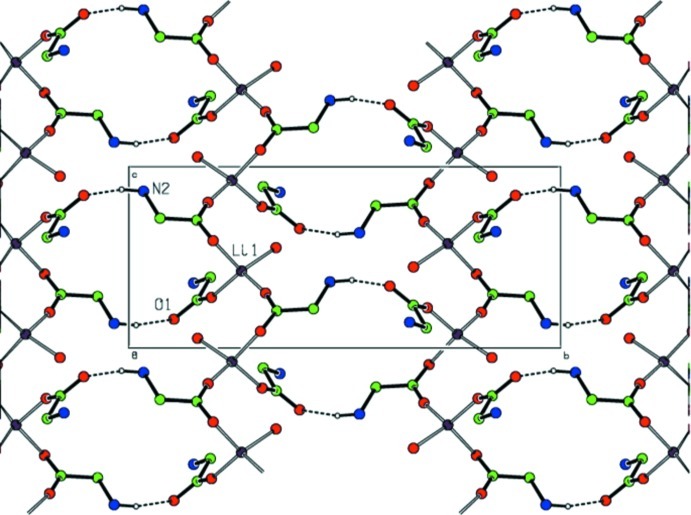
Part of the crystal structure showing N1—H2C···O1 hydrogen bond links the coodination polymeric chains.

**Fig. 5.**
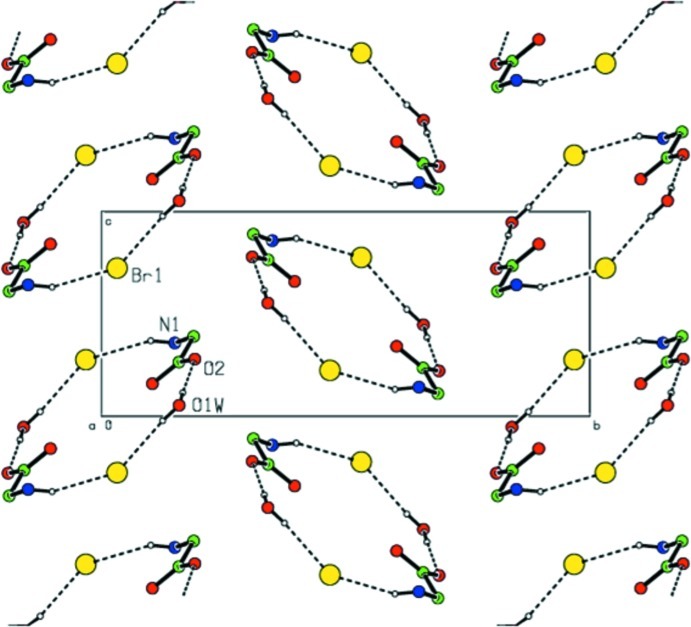
Part of the crystal structure showing R46(18) ring motif which comprises two glycines, two waters and two Br- anions. Hydrogen bonds are indicated by dashed lines.

## supplementary crystallographic information

### Comment

The asymmetric unit of the title complex contains two glycine molecules, one Li cation, one Br^-^ anion and a water molecule (Fig. 1). The bond lengths and angles around the carboxylic groups of both glycine molecules indicate that they are deprotonated and each carboxylic group then carries a negative charge. The amino groups of the glycine molecules are protonated. The positive charge of the ammonium groups are compensated for by the negative charge of the carboxylate groups. The central Li atom is coordinated by a water molecule and three carboxylate oxygen atoms of the three glycine molecules, and has a distorted tetrahedral coordination geometry. One of the two glycine molecules acts as a bridging ligand connecting neighbouring complexes to an infinite chain parallel to the *c* axis (Fig. 2).

The ammonium group of one glycine molecule is involved in an intermolecular hydrogen bond (N1—H1A···O4) with an adjacent glycine molecule (Table 1). Another amino group of the second glycine molecule also participates in an intermolecular hydrogen bond (N2—H2B···O1) with a neighbouring carboxylate group of a glycine molecule. These two hydrogen bonds combined to produce C^2^_2_(10) (Bernstein *et al.*, 1995) chains that run parallel to the *c* axis. Adjacent C^2^_2_(10) chains are connected by another N1···O1 hydrogen bond *via* hydrogen H2C. Two types of N2···O1 hydrogen bonds generate two ring motifs, *R*^2^_4_(8) and *R*^4^_4_(20), with C^2^_2_(10) chains (Fig. 3). These two ring motifs are arranged alternately along the *c* axis.

Each polymer chain is interconnected with neighbouring polymeric chains *via* a hydrogen bond (N2—H2C···O1, Table 1) involving the ammonium group of glycine and a symmetry-related carboxylate group. This hydrogen bond produce two ring motifs *R*^2^_2_(18) and *R*^2^_2_(26). These two rings motifs are arranged alternately along the *bc* plane (Fig. 4). Four glycine molecules and two Li cations are involved in the former ring, while six glycines and four Li ions are involved in the latter motif. The Br^-^ anion acts as an acceptor for two different ammonium groups (atoms N1 and N2) of the glycine molecules. The water molecule acts as a donor for two different intermolecular hydrogen bonds with a carboxylate oxygen (O2) and the Br^-^ anion. The N1—H1C···Br1, O1W—H1···O2 and O1W—H2···Br1 hydrogen bonds held together to form a *R*^4^_6_(18) ring motif (Fig. 5).

### Experimental

A 1:1 stoichiometeric mixture of glycine and lithium bromide was dissolved in double distilled water. Colourless block-shaped single crystals were obtained after 2 weeks by slow evaporation.

### Refinement

The positions of H atoms bound to nitrogen and water oxygen were determined from difference electron density maps and refined freely along with their isotropic displacement parameter. The O—H distances of water molecule are restrained to 0.84 (2) Å using DFIX option. The H atoms bound to carbon were placed in geometrically idealized positions (C—H = 0.99 Å) and constrained to ride on their parent atoms with U_iso_(H) = 1.2U_eq_(C).

### Crystal data

**Table:**

[Li(C_2_H_5_NO_2_)_2_(H_2_O)]Br	*F*(000) = 512
*M**_r_* = 255.01	*D*_x_ = 1.768 Mg m^−^^3^
Monoclinic, *P*2_1_/*c*	Mo *K*α radiation, λ = 0.71073 Å
Hall symbol: -P 2ybc	Cell parameters from 7161 reflections
*a* = 7.5396 (6) Å	θ = 2.3–26.0°
*b* = 17.4173 (14) Å	µ = 4.28 mm^−^^1^
*c* = 8.2726 (12) Å	*T* = 173 K
β = 118.138 (7)°	Rod, colourless
*V* = 957.96 (18) Å^3^	0.61 × 0.30 × 0.30 mm
*Z* = 4	

### Data collection

**Table:**

STOE IPDS diffractometer	1847 independent reflections
Radiation source: fine-focus sealed tube	1520 reflections with *I* > 2σ(*I*)
Graphite monochromator	*R*_int_ = 0.043
phi rotation scans	θ_max_ = 26.0°, θ_min_ = 2.3°
Absorption correction: multi-scan (*MULscanABS* in *PLATON*; Spek, 2009)	*h* = −9→9
*T*_min_ = 0.217, *T*_max_ = 0.277	*k* = −21→21
7515 measured reflections	*l* = −10→10

### Refinement

**Table:**

Refinement on *F*^2^	Secondary atom site location: difference Fourier map
Least-squares matrix: full	Hydrogen site location: inferred from neighbouring sites
*R*[*F*^2^ > 2σ(*F*^2^)] = 0.021	H atoms treated by a mixture of independent and constrained refinement
*wR*(*F*^2^) = 0.051	*w* = 1/[σ^2^(*F*_o_^2^) + (0.0317*P*)^2^] where *P* = (*F*_o_^2^ + 2*F*_c_^2^)/3
*S* = 0.96	(Δ/σ)_max_ = 0.001
1847 reflections	Δρ_max_ = 0.55 e Å^−^^3^
151 parameters	Δρ_min_ = −0.26 e Å^−^^3^
2 restraints	Extinction correction: *SHELXL97* (Sheldrick, 2008), Fc^*^=kFc[1+0.001xFc^2^λ^3^/sin(2θ)]^-1/4^
Primary atom site location: structure-invariant direct methods	Extinction coefficient: 0.0097 (8)

### Special details

**Table:**

Geometry. All s.u.'s (except the s.u. in the dihedral angle between two l.s. planes) are estimated using the full covariance matrix. The cell s.u.'s are taken into account individually in the estimation of s.u.'s in distances, angles and torsion angles; correlations between s.u.'s in cell parameters are only used when they are defined by crystal symmetry. An approximate (isotropic) treatment of cell s.u.'s is used for estimating s.u.'s involving l.s. planes.
Refinement. Refinement of F^2^ against ALL reflections. The weighted R-factor wR and goodness of fit S are based on F^2^, conventional R-factors R are based on F, with F set to zero for negative F^2^. The threshold expression of F^2^ > 2sigma(F^2^) is used only for calculating R-factors(gt) etc. and is not relevant to the choice of reflections for refinement. R-factors based on F^2^ are statistically about twice as large as those based on F, and R- factors based on ALL data will be even larger.

### Fractional atomic coordinates and isotropic or equivalent isotropic displacement parameters (Å^2^)

**Table:**

	*x*	*y*	*z*	*U*_iso_*/*U*_eq_	
O1	0.4191 (2)	0.89555 (8)	0.8413 (2)	0.0210 (3)	
O2	0.1743 (3)	0.80884 (9)	0.7211 (2)	0.0250 (4)	
O1W	0.0182 (3)	0.65924 (9)	0.4468 (2)	0.0218 (4)	
H1	0.075 (4)	0.6666 (14)	0.385 (4)	0.027 (7)*	
H2	0.084 (4)	0.6254 (14)	0.522 (4)	0.043 (9)*	
O3	−0.1525 (3)	0.81668 (8)	0.1971 (2)	0.0270 (4)	
O4	−0.2535 (2)	0.80493 (8)	0.4080 (2)	0.0217 (3)	
N1	0.5965 (3)	0.84975 (12)	0.6419 (3)	0.0205 (4)	
H1A	0.643 (4)	0.8360 (15)	0.559 (4)	0.031 (7)*	
H1B	0.702 (5)	0.8395 (17)	0.755 (5)	0.038 (8)*	
H1C	0.580 (5)	0.899 (2)	0.631 (4)	0.047 (9)*	
N2	−0.2448 (3)	0.96593 (11)	0.1353 (3)	0.0174 (4)	
H2A	−0.129 (5)	0.9644 (14)	0.156 (4)	0.025 (7)*	
H2B	−0.323 (4)	0.9429 (15)	0.027 (4)	0.029 (7)*	
H2C	−0.284 (4)	1.0168 (17)	0.126 (4)	0.035 (8)*	
C1	0.3295 (3)	0.84132 (11)	0.7358 (3)	0.0153 (4)	
C2	0.4092 (3)	0.81116 (11)	0.6103 (3)	0.0174 (4)	
H2E	0.4342	0.7553	0.6307	0.021*	
H2F	0.3062	0.8189	0.4812	0.021*	
C3	−0.2198 (3)	0.84265 (12)	0.2952 (3)	0.0166 (4)	
C4	−0.2679 (4)	0.92762 (12)	0.2840 (3)	0.0204 (5)	
H4A	−0.4078	0.9343	0.2624	0.025*	
H4B	−0.1772	0.9523	0.4025	0.025*	
Li1	−0.0369 (6)	0.7388 (2)	0.5754 (5)	0.0196 (8)	
Br1	0.24155 (3)	0.968012 (12)	0.27597 (3)	0.02208 (9)	

### Atomic displacement parameters (Å^2^)

**Table:**

	*U*^11^	*U*^22^	*U*^33^	*U*^12^	*U*^13^	*U*^23^
O1	0.0197 (8)	0.0202 (8)	0.0247 (9)	−0.0040 (6)	0.0118 (7)	−0.0074 (6)
O2	0.0244 (9)	0.0289 (8)	0.0290 (10)	−0.0120 (7)	0.0186 (8)	−0.0097 (7)
O1W	0.0316 (9)	0.0191 (8)	0.0248 (10)	0.0070 (7)	0.0217 (9)	0.0058 (7)
O3	0.0410 (10)	0.0198 (7)	0.0349 (10)	0.0062 (7)	0.0300 (9)	0.0011 (7)
O4	0.0254 (8)	0.0231 (7)	0.0223 (9)	0.0046 (6)	0.0159 (8)	0.0045 (6)
N1	0.0191 (11)	0.0237 (10)	0.0236 (12)	−0.0025 (8)	0.0142 (11)	−0.0050 (8)
N2	0.0149 (9)	0.0166 (9)	0.0213 (11)	0.0007 (8)	0.0089 (9)	−0.0015 (8)
C1	0.0165 (10)	0.0150 (9)	0.0145 (11)	0.0024 (8)	0.0073 (10)	0.0018 (8)
C2	0.0191 (11)	0.0168 (9)	0.0201 (12)	−0.0012 (8)	0.0123 (10)	−0.0027 (8)
C3	0.0128 (10)	0.0204 (10)	0.0157 (12)	0.0008 (8)	0.0061 (10)	−0.0019 (8)
C4	0.0253 (12)	0.0207 (11)	0.0211 (12)	0.0008 (9)	0.0158 (11)	−0.0023 (9)
Li1	0.023 (2)	0.0190 (16)	0.020 (2)	−0.0040 (14)	0.0125 (18)	−0.0022 (14)
Br1	0.01662 (13)	0.02188 (12)	0.02587 (15)	−0.00224 (9)	0.00846 (10)	−0.00292 (9)

### Geometric parameters (Å, º)

**Table:**

O1—C1	1.247 (3)	N1—H1C	0.86 (3)
O2—C1	1.253 (3)	N2—C4	1.480 (3)
O2—Li1	1.915 (4)	N2—H2A	0.81 (3)
O1W—Li1	1.908 (4)	N2—H2B	0.90 (3)
O1W—H1	0.816 (17)	N2—H2C	0.93 (3)
O1W—H2	0.829 (18)	C1—C2	1.518 (3)
O3—C3	1.228 (3)	C2—H2E	0.9900
O3—Li1^i^	1.880 (4)	C2—H2F	0.9900
O4—C3	1.261 (3)	C3—C4	1.516 (3)
O4—Li1	1.944 (4)	C4—H4A	0.9900
N1—C2	1.472 (3)	C4—H4B	0.9900
N1—H1A	0.94 (3)	Li1—O3^ii^	1.880 (4)
N1—H1B	0.92 (4)		
			
C1—O2—Li1	144.09 (18)	O3—C3—O4	126.0 (2)
Li1—O1W—H1	123.4 (18)	O3—C3—C4	118.73 (18)
Li1—O1W—H2	108 (2)	O4—C3—C4	115.30 (17)
H1—O1W—H2	106 (3)	O3—C3—Li1	96.65 (15)
C3—O3—Li1^i^	169.50 (19)	C4—C3—Li1	134.84 (17)
C3—O4—Li1	116.16 (16)	N2—C4—C3	111.84 (17)
C2—N1—H1A	114.4 (17)	N2—C4—H4A	109.2
C2—N1—H1B	113.0 (18)	C3—C4—H4A	109.2
H1A—N1—H1B	105 (3)	N2—C4—H4B	109.2
C2—N1—H1C	111 (2)	C3—C4—H4B	109.2
H1A—N1—H1C	105 (3)	H4A—C4—H4B	107.9
H1B—N1—H1C	108 (3)	O3^ii^—Li1—O1W	101.73 (17)
C4—N2—H2A	110 (2)	O3^ii^—Li1—O2	116.6 (2)
C4—N2—H2B	110.4 (17)	O1W—Li1—O2	118.6 (2)
H2A—N2—H2B	109 (3)	O3^ii^—Li1—O4	103.87 (18)
C4—N2—H2C	109.9 (18)	O1W—Li1—O4	111.3 (2)
H2A—N2—H2C	109 (2)	O2—Li1—O4	103.93 (17)
H2B—N2—H2C	108 (3)	O3^ii^—Li1—C3	128.00 (19)
O1—C1—O2	125.69 (19)	O1W—Li1—C3	99.35 (16)
O1—C1—C2	118.81 (18)	O2—Li1—C3	92.83 (15)
O2—C1—C2	115.49 (18)	O4—Li1—C3	24.36 (7)
N1—C2—C1	112.13 (17)	O3^ii^—Li1—H2	89.3 (7)
N1—C2—H2E	109.2	O1W—Li1—H2	20.0 (6)
C1—C2—H2E	109.2	O2—Li1—H2	112.4 (8)
N1—C2—H2F	109.2	O4—Li1—H2	130.3 (7)
C1—C2—H2F	109.2	C3—Li1—H2	119.3 (6)
H2E—C2—H2F	107.9		
			
Li1—O2—C1—O1	168.2 (3)	C1—O2—Li1—C3	−72.0 (3)
Li1—O2—C1—C2	−10.9 (4)	C3—O4—Li1—O3^ii^	−172.65 (17)
O1—C1—C2—N1	3.5 (3)	C3—O4—Li1—O1W	−63.9 (2)
O2—C1—C2—N1	−177.3 (2)	C3—O4—Li1—O2	64.9 (2)
Li1^i^—O3—C3—O4	−14.7 (13)	O3—C3—Li1—O3^ii^	−133.0 (2)
Li1^i^—O3—C3—C4	165.1 (10)	O4—C3—Li1—O3^ii^	9.1 (2)
Li1^i^—O3—C3—Li1	14.2 (11)	C4—C3—Li1—O3^ii^	84.0 (3)
Li1—O4—C3—O3	49.0 (3)	O3—C3—Li1—O1W	−20.0 (2)
Li1—O4—C3—C4	−130.8 (2)	O4—C3—Li1—O1W	122.0 (2)
O3—C3—C4—N2	6.3 (3)	C4—C3—Li1—O1W	−163.0 (2)
O4—C3—C4—N2	−173.94 (19)	O3—C3—Li1—O2	99.58 (17)
Li1—C3—C4—N2	143.3 (2)	O4—C3—Li1—O2	−118.4 (2)
C1—O2—Li1—O3^ii^	152.4 (3)	C4—C3—Li1—O2	−43.4 (3)
C1—O2—Li1—O1W	30.3 (4)	O3—C3—Li1—O4	−142.0 (3)
C1—O2—Li1—O4	−93.9 (3)	C4—C3—Li1—O4	75.0 (3)

Symmetry codes: (i) *x*, −*y*+3/2, *z*−1/2; (ii) *x*, −*y*+3/2, *z*+1/2.

### Hydrogen-bond geometry (Å, º)

**Table:**

*D*—H···*A*	*D*—H	H···*A*	*D*···*A*	*D*—H···*A*
N1—H1*A*···O4^iii^	0.94 (3)	1.83 (3)	2.774 (2)	176 (3)
N1—H1*B*···O1*W*^iv^	0.92 (4)	2.15 (4)	2.989 (3)	151 (2)
N1—H1*C*···Br1^v^	0.86 (3)	2.61 (3)	3.353 (2)	146 (3)
N2—H2*A*···Br1	0.81 (3)	2.48 (3)	3.283 (2)	170 (3)
N2—H2*B*···O1^vi^	0.90 (3)	2.00 (3)	2.833 (3)	153 (2)
N2—H2*C*···O1^vii^	0.93 (3)	1.92 (3)	2.797 (2)	157 (3)
O1*W*—H1···O2^i^	0.82 (2)	1.88 (2)	2.692 (2)	172 (3)
O1*W*—H2···Br1^ii^	0.83 (2)	2.48 (2)	3.2923 (17)	169 (3)

Symmetry codes: (i) *x*, −*y*+3/2, *z*−1/2; (ii) *x*, −*y*+3/2, *z*+1/2; (iii) *x*+1, *y*, *z*; (iv) *x*+1, −*y*+3/2, *z*+1/2; (v) −*x*+1, −*y*+2, −*z*+1; (vi) *x*−1, *y*, *z*−1; (vii) −*x*, −*y*+2, −*z*+1.
